# Supplementary material for: Extended 3D-PTV for direct measurements of Lagrangian statistics of canopy turbulence in a wind tunnel
Source: Sci Rep. 2019 May 15;9:7405. doi: 10.1038/s41598-019-43555-2 (PMC6520359; doi:10.1038/s41598-019-43555-2)
Supplement: Supplementary file 1 — Supplementary Material - Extended 3D-PTV for direct measurements of Lagrangian statistics of canopy turbulence in a wind tunnel [file 41598_2019_43555_MOESM1_ESM.pdf]

Supplementary Material - Extended 3D-PTV for direct measurements of Lagrangian statistics of canopy turbulence in a wind tunnel

Ron Shnapp, Erez Shapira, David Peri, Yardena Bohbot-Raviv, Eyal Fattal and Alex Liberzon

The Blob Recorder Algorithm

This section describes in details the basic blob analysis performed on FPGA framegrabber using the library provided by Silicon Software GmbH. For more detailed information an application note from [3] is recommended.

The 8 bit gray scale image is transformed into a black/white (0/1) image. There are two basic binarization methods implemented in the blob recorder. First is a simple thresholding, such that all the pixels with values above a user-selected threshold turn into white (1), and the rest become black (0) and are removed from the following analysis. A more complex adaptive local thresholding is implemented for the cases of non-uniform illumination, for which the background cannot be segmented out using a single threshold.

In the following description, the notation (i, j) refers to pixel location in image coordinates. Column location (x-axis) is i in range $[0, M-1]$, where M is the width, and row location (y-axis) is j in range $[0, N-1]$ where N is the height of the image. $(i, j) = (0, 0)$ refers to the top-left corner of the image.

The algorithm stages depicted below are presented in the same order that they are performed, so generally the output of each stage is fed as an input to the next stage.

- **Streaming input** The recording system is based on four CMOS cameras (Optronis GmbH) that transfer data to the frame-grabber. It uses the CoaXPress (CXP) protocol in 4 lanes, each up to 6.25 Gbit/sec, at a maximal total bandwidth of 25 Gbit/sec. The camera resolution is 2304×1720 ($M \times N$) at frame rates up to 500 Hz (frames per second). All further image processing is performed in real-time on the entire input stream, at full frame rate of the camera. An example of the input image is shown in Fig. 1a (only a small region of 256×256 pixels is shown for the sake of brevity).

- **Input buffer** Image data buffering is preformed with 2 parallel high-speed DDR3 memory banks. Each memory bank is 256 MByte, with data bandwidth of 3.2 GByte/sec. When there is momentary load on image processing engine or on image transfer, the image data is accumulated on the input buffer without losing data.

$$P(i,j)_{\text{out}} = P(i,j)_{\text{in}}$$

- **8-bit Lookup Table (LUT)**: Each 8-bit input grey value is mapped to another 8-bit value, using some pre-defined lookup table. This can be used for performing digital gain or any other arithmetic function on input grey values.

$$P(i,j)_{\text{out}} = \text{LUT}(P(i,j)_{\text{in}})$$

where $\text{LUT}(x)$ is the lookup table's output value for entry x. Both x and $\text{LUT}(x)$ are integer values in range [0, 255]. Default mapping is identity mapping:

$$\text{LUT}(x) = x$$

- **Median Filter** is an optional step that can be turned on/off by the user, where each pixel is replaced by the median of 3×3 neighboring pixels. In our application it helps to remove small noisy areas, typically sized 1-2 pixels.

$$P(i,j)_{\text{out}} = \text{median}\begin{bmatrix} P(i-1,j-1) & P(i,j-1) & P(i+1,j-1) \\ P(i-1,j) & P(i,j) & P(i+1,j) \\ P(i-1,j+1) & P(i,j+1) & P(i+1,j+1) \end{bmatrix}_{\text{in}}$$

- **Background subtraction**: A pre-defined background image can be loaded into this operator. This background image is stored in 2 parallel high-speed DDR3 memory banks. Then for each new frame, the background image is read from memory and subtracted from the input image, pixel-by-pixel. This allows removing of any constant spatial noise and unwanted light reflections from the image scene.

$$P(i,j)_{\text{out}} = \max(0, (P(i,j)_{\text{in}} - P(i,j)_{\text{background}}))$$

- **Offline image injection**: An offline image from pre-recorded file can be injected into the algorithm instead of the camera input image. This can be used for testing a known image with various parameter values, and for adjusting the algorithm parameters. When offline image is injected, the background subtraction cannot be performed, since both operators are sharing the same physical memory banks.

$$P(i,j)_{\text{out}} = P(i,j)_{\text{offline}}$$

- **Polarity Inversion** [optional]: This optional operation inverts each pixel's grey level, for instance in shadow imaging PTV when the system locates dark objects on a bright background. The inversion is required since the following segmentation algorithm is designed to find bright objects on a dark background. This operation can be skipped if not required.

$$P(i,j)_{\text{out}} = 255 - P(i,j)_{\text{in}}$$

- **Binarization**
  - **Local adaptive threshold**: the input image is binarized by comparing each pixel to the average of its surrounding pixels:
    1. First, for each pixel, a local average of the surrounding kernel of a pre-defined size, e.g. 8×8 (presently 8, 16 and 32 pixel^2 kernels are implemented but pixels are diluted to 8×8 values) pixels is computed. A positive offset may be added to the average.

    $$A\left(i,j\right) = \text{Offset} + \sum_{m=i-4}^{i+3} \sum_{n=j-4}^{j+3} P\left(m,n\right)_{\text{in}}$$

    2. Then each pixel value is compared against the value of its local average plus the offset, $A(i,j)$. If the difference is greater or equal to a pre-defined threshold T_{diff}, or pixel value itself is is greater or equal to some absolute threshold T_{fixed}, then the pixel marked as bright pixel (1). Otherwise the pixel is marked as dark pixel (zero or background).

    $$P(i,j)_{\text{in}} - A(i,j) \geq T_{\text{diff}} \vee P(i,j)_{\text{in}} \geq T_{\text{fixed}} \Rightarrow P(i,j)_{out} = 1$$

  - **Fixed threshold**: An alternative image binarization method compares each pixel to some pre-defined fixed threshold, T_{fixed}:

  $$P(i,j)_{\text{in}} \geq T_{\text{fixed}} \Rightarrow P(i,j)_{out} = \ 1$$

- **Image output**: There is an option to store the output image P_{out} to the host PC memory for display or further off-line image processing. In order to decrease system load, the operator can output N image in range [1, 511]. For debugging purposes the output image can be stored before or after binarization stage (converted from [0,1] to [0,255] range). An example of the binarized image is shown in Fig. 1b.
- **Segmentation using blob analysis**: A rectangular region of interest (ROI) from the binary image is passed to segmentation and blob analysis. Note that if necessary the image resolution can be reduced, which then allows a significant increase of the data acquisition rate (see Fig. 1).

Blob analysis algorithm locates and measures connected regions of bright pixels (called blobs), and outputs information on each one of the connected regions for further analysis of the scene. The data output on each blob includes area in pixels, center of gravity coordinates and enclosing rectangle coordinates. The center of gravity coordinates are given at resolution of 1/256 pixel. All coordinates are relative to image top-left corner. The current implemented algorithm uses 8-connected objects, but it can be easily modified to find 4-connected objects. Detailed information on implementation of blob analysis operator in FPGA is provided by the framegrabber manufacturer [3]. The result can be displayed on an original image as marked blobs, see Fig. 1c.

- **Objects filtering and output**: The blobs are optionally filtered by area and only blobs in range [area_{min}, area_{max}] are stored to the host PC. This stage can remove very small objects, which can be considered as noise, and also very large objects, such as background light reflected on image.

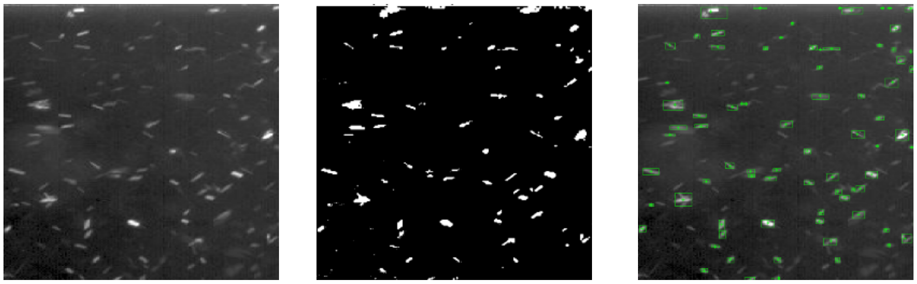

(a) (b) (c)

Figure 1: An example of a raw 3D-PTV image from laboratory experiment [2]. (a) Original image example, (b) binarized image, (c) blobs marked on the original image. This example is using median filter, without polarity inversion, an adaptive filter with the offset of 20, $T_{\text{diff}} = 0$, $T_{\text{fixed}} = 90$ and filtering out blobs smaller than 20 pixels.

The data compression ratio is significantly larger than typical real-time image processing routines for PIV or PTV [2]. At 25 Gbit/sec the input stream can be recorded to the RAM of the host PC for several seconds. Although this is a possible solution for short recordings, calibration or adjustment of parameters (saving offline or background images), the wind tunnel measurements require continuous recording to enable tracking at full speed and intermittently when the seeding creates an acceptable particle images quantity and quality. The processed data of blobs is stored at roughly $\sim 1/1000$ compression ratio.

Benchmark of Real-Time Detection Methods

Performance of Detection Algorithms

The main advantage of the proposed extension is the ability to perform complex image analysis on hardware in real time. This feature allows performing 3D-PTV experiments in situations that involve complex background illumination, reflections and noisy images, as was demonstrated for the canopy flow experiment. In previous real-time solutions [1, 2], particles were identified using binary image segmentation, with a global brightness threshold or by a predefined global edge detection threshold. In order to compare the performance of the image analysis algorithms, starting from the binary segmentation to the implemented algorithm with increasing complexity, we perform a benchmark using synthetic particle images.

The synthetic images benchmark contains a particle image layer, comprising 151 white "particles" of diameter either 1, 4, or 8 pixels. The particle images were overlaid using two different backgrounds of increasing complexity. The first one is a uniform black background (Fig. 2(a)) simulating an ideal imaging condition, or physically possible 3D-PTV experiment, with the tracers scattering or emitting light with different illumination. The second background image (Fig. 2(b)) incorporates three possible physically relevant components - a brightness gradient across the entire image, six spots of Gaussian intensity, and a weak random noise ("Hurl"). The benchmark of three different detection algorithms,

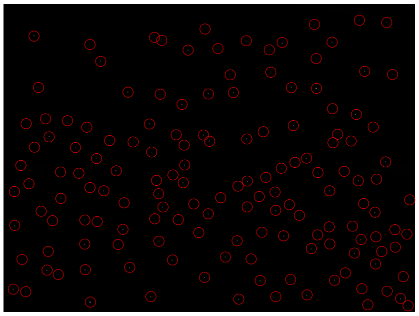

(a)

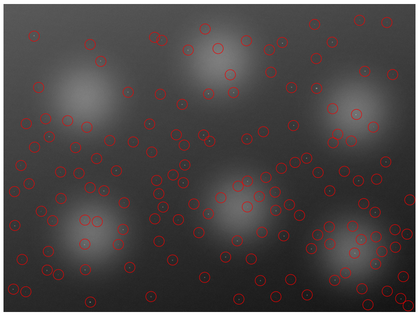

(b)

Figure 2: Synthetic Benchmark images (a) particles over uniform black background (b) particles over a complex, unevenly illuminated and noisy, background. Red circles mark the positions of particles.

co-existing in the presented hardware solution, was assessed using these two images. For the comparison we used two dimensionless quality parameters:

$$Q_T = \frac{T}{N_p} \times 100\% \quad (1)$$

$$Q_F = \frac{T}{T+F} \times 100\% \quad (2)$$

where N_p is the number of "real" particles in the image, T is the number of particles that were detected out of the real N_p particles (i.e. true positive), and F is the number of wrongfully marked particles (i.e. false positive). In the perfect segmentation scenario, all and only the real particles will be detected such that $Q_T = Q_F = 100\%$.

The Benchmark was performed as follows - a particle detection algorithm was used to segment the particles in each of the two images. In each case, the global brightness threshold for segmentation was tuned until the maximal amount of particles is found while no false negatives are detected $Q_F = 100\%$; this corresponds to the practical case of complete intolerance to false detections. The value of Q_T was then marked and later used for comparison between algorithms. The three algorithms tested were i) the binary segmentation that searches for global peaks of brightness ii) the adaptive threshold that searches for local peaks of brightness compared to the local neighbourhood and iii) background removal with adaptive threshold, where a static image is subtracted from the particle image and an adaptive threshold algorithm is used to detect the local brightness peaks. Fig. 3 presents the results of the test. For the ideal image case, all three algorithms were equally successful in identifying all the particles in the image - $Q_T = 100\%$. However, in the complex background case, the binary segmentation only detected $Q_T \approx 20\%$ of the particles. The adaptive threshold algorithm was able to retrieve roughly triple than the former $Q_T \approx 60\%$, and only after applying background removal $Q_T \approx 100\%$ of the particles were detected. Applying background removal on the complex background image left only the random noise, thus reducing the detection task of the adaptive threshold to a nearly ideal image case. Thereby we conclude that while simple binary segmentation is good for particle detection in ideal imaging conditions, noisy images with uneven background illumination require a more complex algorithm for the successful detection of all particles in the image with no false detections.

Computational Costs

The time of computation required by the detection algorithm sets a limit for the rate at which real-time detection solutions can be applied for the extended 3D-PTV. In addition, the more complex algorithms demand more computational resources and thus come at higher computational costs. For example, Table 1 shows the computational time needed for applying each algorithm and the maximal frame rate available over 4 Megapixel images, with the FPGA hardware used in the current BlobRecorder (Xilinx Virtex6 XC6VLX240T). It should be noted that in the current application, the acquisition rate was limited by the maximal data rate available using the CoaXPress standard (2.4Gb/s). A possible extension can be achieved either by using cameras with multiple CoaXPress channels or by using a faster variant of the CoaXPress standard with compatible cameras.

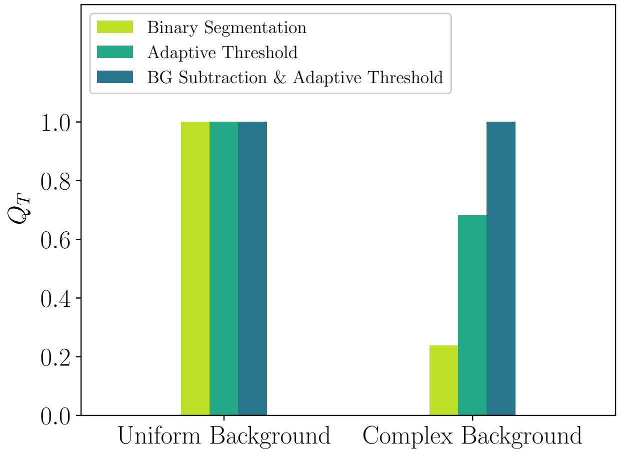

Figure 3: Benchmark of particle detection showing the Q_T quality parameter obtained for the two synthetic images, such that $Q_F = 100\%$ implying intolerance to false detections.

Algorithm	Max. data rate	Max. frame rate @ 4MP
Binary Segmentation	8 Gb/s	2000 Frame/sec
BG Subtraction & Binary Segmentation	7 Gb/s	1750 Frame/sec
Adaptive Threshold	4 Gb/s	1000 Frame/sec
BG Subtraction & Adaptive Threshold	2.5 Gb/s	625 Frame/sec

Table 1: Computational cost of each detection algorithm performed on the used FPGA hardware.

Raw experimental image

An example of a raw image from the wind tunnel canopy experiment is shown in Fig. 4. The laser light reflects strongly from the canopy roughness elements and the wind tunnel bottom wall. Since a binary segmentation of such images cannot adequately extract tracer particles' positions, the complex image analysis algorithm was needed in this case.

Some of the tracers in the image, depending on their speed, appeared as streaks, yet the image analysis algorithm was able to detect them. This may affect the possibility of extending such measurements to higher seeding densities due to an apparent increase in image density. A strobing illumination source may be considered as a solution to this problem.

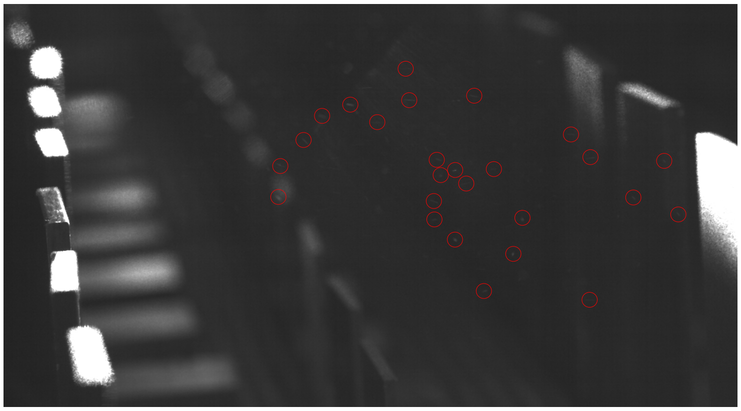

Figure 4: An experimental raw image at $z \approx 1.25H$, and $U_\infty = 4$ m/s. Tracer particles are highlited against the background manually with red circles.

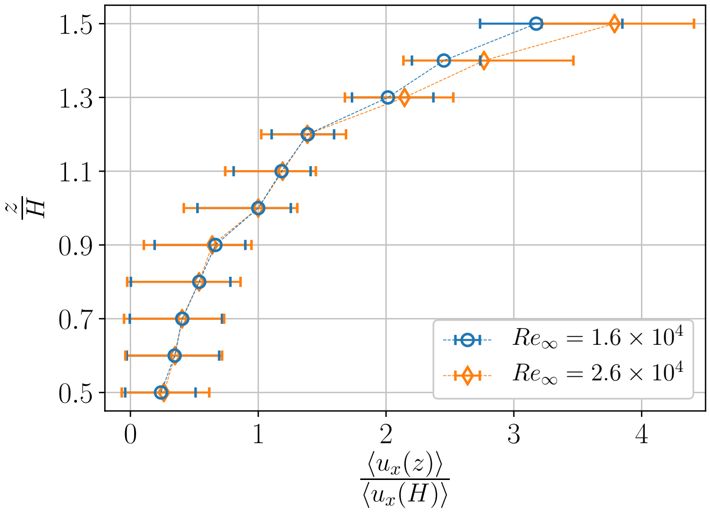

Figure 5: Horizontally averaged mean velocity profile. The velocity is normalized by the mean value at the top of the canopy layer $z = H$. The error bars represent the minimum / maximum single point averaged velocity values at each height.

Mean Velocity Profile

In Fig. 5 we present the horizontally averaged mean streamwise velocity as a function of height. In order to produce velocity statistics over an Eulerian grid, the observation volume was divided into cubes of size $1 \times 1 \times 1$ cm^3. At each

cube, we calculated the average of the velocity samples belonging to particles that passed through it at the times it was inside the cube. Following that we calculated the horizontally averaged velocity by averaging the values of mean velocity found at the same height. The error bars in Fig. 5 describe the spatial variability of the mean velocity profile by showing the minimum and maximum values of averaged velocity found at each height. It can be seen that the mean velocity profiles from two Re_∞ collapse almost perfectly inside and slightly above the canopy, whereas they deviate from each other further high up ($z >\sim 1.2H$).

References

[1] K.-Y. Chan, D. Stich, and G. A. Voth. Real-time image compression for high-speed particle tracking. *Review of Scientific Instruments*, 78(2):023704, 2007.

[2] M. Kreizer and A. Liberzon. Three-dimensional particle tracking method using fpga-based real-time image processing and four-view image splitter. *Experiments in Fluids*, 50(3):613–620, 2011.

[3] SiliconSoftware. *Blob Analysis Operators, Object Detection on FPGA Hardware, User Manual.* SiliconSoftware GmbH, 2.0 edition, may 2013.
